# Management of Proximal Humerus Fractures in Adults—A Scoping Review

**DOI:** 10.3390/jcm11206140

**Published:** 2022-10-18

**Authors:** Hayden P. Baker, Joseph Gutbrod, Jason A. Strelzow, Nicholas H. Maassen, Lewis Shi

**Affiliations:** The University of Chicago, Department of Orthopaedic Surgery and Rehabilitation Medicine, The University of Chicago Hospitals, Duchossois Center for Advanced Medicine, MC 3079, 5758 S. Maryland Avenue, Dept 4B, Chicago, IL 60637, USA

**Keywords:** proximal humerus, fracture, management, shoulder arthroplasty

## Abstract

Proximal humerus fractures are the third most common fracture type in adults, with their incidence increasing over time. There are varied approaches to both the classification and treatment of proximal humerus fractures. Optimal treatments for this fracture type are still widely open to debate. This review summarizes the current and historical treatment modalities for proximal humerus fractures. In this paper, we provide updates on the advances and trends in the epidemiology, classification, and operative and nonoperative treatments of proximal humerus fractures.

## 1. Epidemiology

Proximal humerus fractures represent 5–6% of all fractures [1,2]. It is the third most common fracture in older adults [3]. The incidence of proximal humeral fractures varies by region. An analysis in southern Europe showed an incidence rate of 60.1 proximal humeral fractures per 100,000 person-years from 2016 to 2018 [4]. In Australia, the incidence of proximal humeral fractures was found to be 45.7 fractures per 100,000 person-years in 2017 [5].

Females sustain higher rates of proximal humeral fractures than males [1,4,5]. In the southern Europe analysis, the female incidence rate of proximal humeral fracture was 89.3 per 100,000 person-years as compared to 28.2 in men [4]. In Australia, women over 85 years old had the highest proximal humeral fracture incidence at 711.8 fractures per 100,000 person years in 2017 [5]. Typically proximal humerus fractures occur in a bimodal distribution pattern; high energy injuries in young individuals and low energy injuries in elderly individuals with osteoporotic bone [1].

Proximal humeral fracture incidence is also increasing over time. In Australia, proximal humeral fracture incidence rose from 28.5 per 100,000 in 2008 to its 2017 rate of 45.7 per 100,000 [5]. This increasing trend has been occurring for the last several decades, with the population-adjusted incidence rate in New York City rising from 15.35 per 100,000 in 1990 to 19.4 per 10,000 in 2010 [6]. The increasing trend in PHF incidence is most likely due to the increasing median age of the population in these studies (Khatib et al., 6th citation below).

## 2. Classification

An early classification of proximal humerus fractures was created in 1934 by Ernest Codman, who defined fractures based on involvement of four anatomic parts: humeral shaft, articular surface, greater tuberosity, and lesser tuberosity [7]. The Codman classification, however, did not consider fracture displacement or differentiate between surgical and anatomic neck fractures [8].

The Neer classification system, published in 1970, is the most commonly used classification system for proximal humerus fractures [9]. Neer expanded upon Codman’s use of the four main anatomic segments, while also quantifying and qualifying fracture displacement [8]. He defined displacement as a fracture segment separated more than 1 cm or angulated more than 45 degrees. Using this definition of displacement, Neer defined fractures as one-part, two-part, three-part, or four-part. Under this system, all non-displaced fractures are grouped together while displaced fractures require more specific groupings. Neer used this approach to further classify fractures into six groups: I: minimal displacement; II: displaced anatomic neck fracture; III: displaced surgical neck fracture; IV: displaced greater tuberosity fracture; V: displaced lesser tuberosity fracture; and VI: fracture-dislocation. An analysis by Court-Brown et al. found that, under the Neer system, 49% of proximal humerus fractures fall under group I: minimally displaced [1]. Despite the classification’s wide adoption, its overall reliability remains fair to moderate in both intra- and inter-observer reliability (K = 0.2–0.8) [10].

The AO/OTA classification, developed in the 1980s, classifies proximal humerus fractures based on articular surface involvement, anatomic location, and dislocation [11]. This system divides PHFs into three main types: Type A-unifocal extra-articular; Type B-bifocal extra-articular and Type C-articular. These types are further subdivided into 27 total subgroups. Importantly, the OTA classification identifies the importance of valgus impaction of the proximal humeral neck which is a distinct fracture pattern not included in previous classification systems.

In 2004, Hertel et al. developed a ‘lego-block’ binary classification system, the Codman-Hertel system, to help identify predictors of fracture-induced humeral head ischemia [12]. Specifically, Hertel’s group identified the importance of fracture morphology specifically; disruption of the medial hinge and extension of the medial calcar (<8 mm) as important predictors for fracture fragment and head vascularity. This system, also building off Codman’s early work, utilized structured questionnaires to divide proximal humerus fractures into 12 basic types. Resch et al. created a similar classification scheme that focused largely on valgus vs varus impacted fractures [13,14].

The inter-rater reliability of proximal humeral fracture classifications was calculated by Majed et al., who found that Codman-Hertel had the highest inter-rater reliability, followed by Neer, then Resch, and finally AO/OTA [15].

## 3. Clinical and Radiographic Assessment

The surgeon must perform a focused detailed history and physical examination of patients with a proximal humerus fracture, paying particular attention to the mechanism of injury and patient-related factors that may have contributed to the fracture. The patient’s activity level, handedness, and living situation should also be assessed to determine future activity needs. Additional points to obtain on history include the presence or absence of prior shoulder trauma or rotator cuff dysfunction as these may alter available treatment options. Associated injuries, particularly amongst elderly or comorbid patients, include head injuries and concomitant extremity fractures.

Neurovascular injury is another significant concern during clinical evaluation of proximal humerus fractures [16,17,18]. Surgeons should check for injury of the axillary nerve, brachial plexus, and distal circulation with special attention to active firing (isometric contraction is sufficient) of the deltoid [16]. Doppler ultrasound and Computerized Tomographic Angiography (CTA) are used to assess for vascular injury [17]. If the patient has hard signs of vascular injury (pallor, pulselessness, paresthesia, paralysis, pulsatile bleeding and or a large or expanding hematoma) then an arterial brachial index (ABI) and/or CTA is recommended. Regarding nerve injury, Visser et al. used electromyography to quantify the incidence of nerve lesion in proximal humerus fractures [19]. They found that 67% of patients exhibited nerve denervation following a PHF. The axillary, suprascapular, and radial nerves were the most affected. Open fractures should receive antibiotic prophylaxis promptly upon admission and are indicated for operative irrigation and debridement. Similarly, ballistic proximal humerus fractures should also receive antibiotic prophylaxis.

A complete orthogonal plane view X-ray series provides helpful information for the typical displacement associated with proximal humerus fracture [16]. The standard radiographic imaging of proximal humerus fractures includes true anteroposterior (Grashey), scapular Y, and axillary views [16,17,18,20]. The axillary or scapular Y views will show common posterior displacement of the greater tuberosity and confirm congruence of the glenohumeral joint [20]. If traditional axillary radiographs cannot be easily obtained due to patient tolerance then ‘trauma axillary’ or velpeau views may offer more tolerable alternatives. The AP and lateral views show the medial displacement of the lesser tuberosity and shaft [20]. When fracture lines cannot be clearly delineated in more complex fracture patterns, then computed tomography imaging is recommended [17,18]. Two-dimensional CT scans are helpful in showing the condition of the articular surface of the humeral head and the glenoid. It can also help visualize the position of the tuberosities [20]. Transthoracic views can sometimes show angulation, as in surgical neck fractures [20].

MRI imaging is not often indicated for evaluating acute proximal humerus fractures, but can be used to assess rotator cuff injury after fracture and for diagnosing non-displaced greater tuberosity fractures [16,17,18,20].

In a more global sense proximal humerus fractures, in elderly individuals, are often considered fragility fractures. Treating surgeons should be recommending vitamin D supplementation for all patients presenting with an acute proximal humerus fracture and considering bone health referrals.

## 4. Treatment Overview

There is debate concerning the ideal treatment options for proximal humerus fractures [16,17,18,21,22]. A Cochrane review of 23 randomized trials and 1238 patients found that there was insufficient evidence to inform the treatment of these fractures [23]. Injury classification, age, and medical history all play a role in the surgeon’s treatment decision [16]. Informed, and patient specific discussions regarding the breadth of treatment options matched to specific patient injury and circumstances as well as surgeon technical expertise is critical.

## 5. Non-Operative Management

The majority (around 65 to 85 percent) of proximal humerus fractures are treated nonoperatively [3,5]. Generally, nonoperative treatment is recommended for stable, minimally displaced fractures or those not deemed surgical candidates [16,24,25].

Nonsurgical management is recommended if all five of the following criteria are met [26,27]:

1. There is contact or impaction between the head and shaft
2. The humeral head is not dislocated
3. There is minimal varus or valgus angulation of the humeral head (head shaft angle between 100 and 160 degrees)
4. There is minimal displacement of any tuberosity fracture
5. There is minimal articular surface involvement.

Common proximal humeral fracture patterns that heal well with nonoperative management include Neer one-part humeral neck fractures [27,28,29], one-part lesser or greater tuberosity fractures [30], and impacted two-part surgical neck fractures with minimal angulation of the head fragment [27]. Displaced Neer two-, three-, and four-part fractures with minimal varus or valgus angulation of the head fragment and with residual cortical contact with the shaft fragment have been shown to heal successfully with nonoperative management [27,31].

The common nonoperative approach is sling immobilization followed by early and progressive physical therapy rehabilitation [16,18,25]. Immobilization options include a standard sling, collar and cuff, shoulder spica cast, hanging arm cast, and airplane splint although these later options are largely historical [16]. The importance of early rehabilitation was shown by Koval et al., who found that patients who started therapy within 14 days of the fracture had significantly better outcomes (based on pain, function, and range of motion) than those who began rehab on or after the two week mark [25]. 

Fractures with tuberosity involvement with displacement of the greater tuberosity or lesser tuberosity are generally strongly considered for surgical management as the risk of further displacement and functional loss may occur. [32]. Unlike humeral shaft fractures, functional bracing is not an effective option for proximal humeral fractures, as they often have complex deforming forces that cannot be neutralized through an external brace [32,33]. However, gravity alone can “reduce” or improve alignment of proximal humerus fractures treated with a trial of conservative management and a sling.

As with all proximal humerus fracture literature, there is debate around the outcomes following nonoperative treatment on proximal humerus fractures, with differences existing between different fracture types and studies [16,24,34,35,36]. Court-Brown et al. analyzed outcomes for 125 patients with a valgus impacted fractures, all of them receiving nonsurgical treatment [24]. They found that 80.6 percent of these patients achieved a good or excellent result. However, Hanson et al. found that the affected shoulders of non-operatively treated proximal humeral fracture patients exhibited significantly lower function than the nonaffected, contralateral shoulder at 1 year follow-up [34]. Functional outcomes in this study were measured by Constant score and the Disabilities of the Arm, Shoulder, and Hand (DASH). Similarly, a meta-analysis by Misra et al. found that nonoperatively managed PHF patients had more pain and a poorer range of motion than those managed by either fixation or arthroplasty [35]. Likewise, a randomized trial by Olerud et al. found that nonoperative treatment led to worse functional outcome and worse quality of life scores than locking plate synthesis for three-part proximal humerus fractures in the elderly [36]. 

Overall, the literature suggests that there may be no significance difference in outcomes between operative and nonoperative treatment, especially for more displaced fractures in elderly individuals (>65 years). The PROFHER trial, the single largest randomized pragmatic trial of proximal humerus fractures, found that, for patients with displaced proximal humeral fractures involving the surgical neck, there was no significant difference between operative treatment versus nonoperative treatment in patient-reported clinical outcomes 2 years post-fracture [37]. In a meta-analysis of 7 randomized controlled trials and 15 observational studies, Beks et al. further confirmed that the functional outcome did not differ between operative and nonoperative treatments [21]. The effectiveness of nonoperative treatment versus operative methods is still widely up to debate.

### Complications

Complications of nonoperative treatment include osteonecrosis, nonunion, malunion, stiffness, and rotator cuff dysfunction [16,34]. Hanson et al. found that, out of 160 nonoperatively managed proximal humeral fracture patients, 7% showed delayed union or nonunion [34]. Total immobility for more than 2 weeks post-injury leads to increasing rates of stiffness for nonoperative treatment [25]. The PROFHER trial found no significant difference in complication related to proximal humerus fracture or its treatment when comparing surgical versus nonsurgical treatment of proximal humeral fractures [37].

## 6. Operative Management

Three factors should be considered during the joint decision-making process to select the optimal treatment for a specific patient: (1) the patient’s physiological status, (2) the severity of fracture pattern, and (3) the experience and skillset of the treating surgeon.

1. Patient Factors

The majority of patients with proximal humeral fractures are elderly with low functional expectations. Thus, operative fixation may be rarely indicated for proximal humerus fractures occurring in elderly or frail patients, patients with significant cognitive impairment, or severe medical comorbidities. Several medical comorbidities have been associated with poor outcomes and increased risk of complication following operative fixation including severe osteoporosis, diabetes mellitus, immunocompromised status, chronic steroid use, concurrent neoplasm, tobacco use, alcohol and illicit drug use, and rheumatoid arthritis [38,39,40].

2. Fracture Severity

Current fracture classification systems serve the surgeon poorly in determining whether operative treatment should be recommended. The Neer classification system remains the most widely used in both research and clinical practice [40], but controversy exists regarding its reproducibility and inter-observer reliability [41]. For example, the number of fracture parts does not necessarily dictate treatment, as most impacted and stable fractures will be treated nonoperatively, regardless of the number of fracture parts [42,43,44,45].

Operative fixation should be discussed with patients with proximal humeral fractures if they are a surgical candidate, have significant displacement, understand the risks inherent to surgical treatment, and in patients that do not meet the criteria for nonoperative management. In patients deemed surgical candidates, fixation technique is largely based on the fracture pattern and bone quality. There is a small group of patients where operative intervention is more directly indicated. These include fracture dislocations, head splitting fractures, fractures with associated vascular injury, and open fractures.

3. Surgeon Factors

The surgeon can significantly influence a patient’s outcome both with the treatment provided and the level of skill with which the treatment is implemented. Technical expertise varies in accordance with a surgeon’s skill set and experience treating these fractures [46]. Additionally, training and proficiencies across techniques have been shown to influence outcomes and preference of treatment [47].

### 6.1. Fractures with a Relative Indication for Operative Fixation

Operative fixation of proximal humeral fractures should be performed with the goal of improving functional outcomes when compared with nonoperative treatment. Inevitably patients who have the most to gain from surgery are typically healthy and physiologically young, however, these are not the typical demographic for this fracture pattern. Avoiding the potential complications of treatment for proximal humerus fractures is paramount. AVN, a devastating complication following the management of shoulder fractures is commonly debated as to whether open reduction and internal fixation decreases the risk of developing osteonecrosis of the humeral head. However, shoulder arthroplasty to treat humeral head collapse secondary to AVN is typically technically easier if the humeral head and tuberosities were anatomically fixed primarily.

Several authors have described proximal humeral fracture patterns that meet relative indications for operative fixation, they include the following:

- Neer two-part surgical neck fractures with complete displacement of the humeral head relative to the shaft or incomplete displacement but no cortical continuity due to comminution [27]
- Neer two-, three-, or four-part greater or lesser tuberosity fracture where the tuberosity if displaced more than 5 mm [26,27]
- Displaced articular surface fracture with articular incongruity or more than 2 mm [26]
- Neer two-, three-, or four-part fracture with greater than 30 degrees varus or valgus angulation of the humeral head relative to the anatomic head shaft angle [26]
- Neer three- or four-part anterior fracture dislocation with retained soft tissue attachments to the humeral head [48]
- Neer three- or four-part posterior fracture dislocation with retained soft tissue attachments to the humeral head [44].

### 6.2. Fractures with a Relative Indication for Arthroplasty

These injuries make up a small minority of all proximal humeral fractures. Fractures that meet criteria for primary arthroplasty include unreconstructable head-splitting fractures, fractures devoid of all soft tissue attachments [48], severe valgus impacted fractures with disruption of the medial periosteal hinge [12,48,49], and displaced Neer multi-part fractures with delayed presentation (greater than 4 weeks from injury).

## 7. Closed Reduction and Percutaneous Pinning

Although open reduction and internal fixation of proximal humerus fractures may result in an anatomic reduction with stable fixation, significant exposure is often required [41]. Minimally invasive techniques, including closed reduction and percutaneous pinning, may offer advantages of less soft tissue stripping over conventional open fixation [41]. Reported advantages of CRPP compared with ORIF include lower rates of avascular necrosis (AVN), higher union rates, decreased scar formation, and improved cosmesis [41]. However, utilization of percutaneous techniques has fallen out of favor recently with the majority of shoulder and trauma surgeons preferring open reduction internal fixation in young patients where surgery is indicated [47].

### 7.1. Indications

Fracture patterns that are amenable to CRPP include:

(1) Two-part fractures
  (a) Greater Tuberosity
  (b) Lesser Tuberosity
  (c) Surgical Neck
(2) Three-part surgical neck fractures with involvement of either the greater or lesser tuberosity
(3) Valgus-impacted four-part fractures.

Generally, head-split fractures and fractures of the anatomic neck are not considered for percutaneous fixation. 

Closed reduction and percutaneous pinning of proximal humerus fractures is a technically challenging procedure that can be successful if the following five conditions are met:

(1) Good bone quality
(2) Minimal comminution
(3) Stable closed reduction
(4) Intact medial calcar
(5) Cooperative patient.

If acceptable length, alignment, and rotation cannot be obtained closed, then the technique should be abandoned in favor of open reduction and internal fixation. Calvo et al. demonstrated that clinical results are correlated with quality of reduction following fixation of proximal humerus fractures [50]. Thus, there is minimal benefit of limited exposure if the fracture cannot be appropriately reduced. Prior to draping the patient, the surgeon can attempt an initial closed reduction under fluoroscopy to determine the likelihood of obtaining an acceptable reduction closed. The final decision regarding fixation construct should be made by the surgeon intra-operatively, however if attempting CRPP the surgeon should have appropriate instruments available should ORIF or arthroplasty be required.

### 7.2. Contraindications

Relative contraindications to CRPP of proximal humerus fractures includes poor bone quality and fracture comminution [41]. CRPP depends on limited fixation with good bone purchase. Loss of reduction and pin loosening has been attributed to poor bone quality and fracture comminution in multiple studies [41,50,51,52].

## 8. Surgical Approach

### 8.1. Patient Positioning

Percutaneous fixation can be performed with the patient positioned in either the supine or beach-chair position. Care should be taken to position fluoroscopy to allow for complete visualization of the proximal humerus in two orthogonal views.

### 8.2. Fracture Reduction

The ability to obtain an anatomic reduction closed is the key to minimally invasive percutaneous techniques. Understanding the deforming forces on the fracture fragments and utilizing reduction maneuvers that counteract the deforming forces can help achieve an anatomic closed reduction. Deforming forces on the fracture fragments are specific to each fracture pattern, thus fracture-specific reduction maneuvers have been previously described [41].

#### 8.2.1. Two-Part Greater and Lesser Tuberosity Fracture

Percutaneous fixation is appropriate for mobile greater tuberosity fractures with minimal displacement. Placement of a percutaneous pin into the tuberosity to act as a joystick, followed by rotation of the humerus, can help reduce the tuberosity. Once reduced, a second pin can be driven through the tuberosity across the fracture site for definitive fixation. Alternatively, a mini-open reduction through a small 2 to 3 cm incision distal to the anterolateral acromion with a deltoid split can be utilized to introduce a reduction pick or bone tamp to assist with tuberosity reduction and fixation. Lesser tuberosity fractures are rarely fixed percutaneously as it can be difficult to obtain an acceptable closed reduction and there is danger of injuring the axillary nerve and anterior humeral circumflex vessels [41].

#### 8.2.2. Two-Part Surgical Neck Fractures

The two major fracture fragments in a two-part surgical neck fracture are the humeral head and the humeral shaft. Typically, the humeral head remains in neutral because all of the rotator cuff attachments to the head fragment are intact. While the pectoralis major muscle displaces the humeral shaft fragment anteromedially and internally rotates it. The pectoralis major is the primary deforming force, and its effects can be minimized by flexing, adducting, and internally rotating the humerus. Traction can then be applied to the arm with a posteriorly directed in order to reduce the apex anterior angulation. Once reduced, the humeral shaft can be externally rotated to the neutral position.

#### 8.2.3. Three-Part Greater Tuberosity Fractures

Similar to two-part surgical neck fractures, the humeral shaft will assume a position of anteromedial displacement and internal rotation secondary to the pull of the pectoralis major. The unopposed pull of the subscapularis on the intact lesser tuberosity internally rotates the head fragment. Reduction of the surgical neck is performed as described above. The surgical neck portion of the fracture is then percutaneously fixed to the humeral shaft. Following fixation, the arm can be positioned in slight external rotation to counteract the pull of the rotator cuff muscles on the greater tuberosity. The tuberosity can then be reduced using either a k-wire or bone hook and fixed with cannulated screws or antegrade pins.

#### 8.2.4. Three-Part Lesser Tuberosity Fracture

Again, the humeral shaft is displaced anteromedially with slight internal rotation secondary to the pectoralis major; while the humeral head is abducted and externally rotated, with apex-anterior angulation due to the unopposed pull of the rotator cuff on the greater tuberosity. Reduction of the surgical neck can be achieved by flexing and externally rotating the arm. While traction and a postero-medially directed force help reduced the humeral shaft to the head component. Often a closed reduction cannot be achieved and a small incision must be made to improve exposure of the fracture fragments. Internal rotation of the arm and Kirschner wires used as joysticks can aid in reduction of the lesser tuberosity.

#### 8.2.5. Four-Part Valgus-Impacted Fracture

In order to obtain an anatomic reduction of a four-part valgus-impacted fracture pattern, the lateral aspect of the humeral head must be elevated. A 2 to 3 cm incision can be made 2 cm distal to the anterolateral acromion to aid with reduction attempts. Typically, a small bone tamp or periosteal elevator can be inserted through the tuberosity defect to engage the lateral aspect of the humeral head and used to disimpact the articular surface from the shaft. The goal of the reduction is to elevate the lateral aspect of the articular surface utilizing the intact medial periosteum as a hinge. Once elevated the head can be percutaneously fixed in that position and then the shaft can be reduced to the head fragment as described above.

### 8.3. Fracture Fixation

Fixation of the humeral shaft to the head is typically performed with 2.5-mm Schantz pins placed in a retrograde fashion from the shaft into the humeral head. Care should be taken to place the pins on the lateral cortex in a safe zone that avoids injury to both the axillary and radial nerves. If the pins are kept above the deltoid insertion, the radial nerve is typically protected. While the axillary nerve is, on average, located 5 cm distal to the acromion [53]. A soft tissue sleeve should be utilized when placing pins through the deltoid to decrease the risk of neurovascular injury. Average humeral retroversion is 19 degrees, thus percutaneous pins should be directed posteromedially to account for this angle [54]. Pins placed from anterior to posterior place the musculocutaneous nerve and biceps tendon at risk and should be avoided if possible.

After fixing the humeral head to the shaft, the tuberosities can be addressed. Cannulated screw and pin fixation have been described for fixation of the tuberosities [55,56]. Under fluoroscopic guidance cannulated screws or pins are typically placed from proximal lateral to distal medial [57]. In order to reduce the risk of pin migration, terminal threads can be utilized to secure pins in place. Care should be taken to advance pins to engage dense subchondral bone. Some authors routinely bend pins beneath the skin to act as a mechanical block to backing out, however migration may still occur.

### 8.4. Postoperative Management

Typically, the operative extremity is immobilized in a sling for the first two weeks postoperatively. Immediate active elbow, wrist, and hand range of motion is permitted. Pendulum exercises can be started immediately, if fixation is thought to be stable enough to tolerate. Pins are removed at 4 to 6 weeks postoperatively when there is radiographic evidence of healing. Plain films should be obtained weekly to ensure that fracture reduction is maintained and pin migration has not occurred. Following pin removal, active-assisted range of motion can be initiated followed by strengthening exercises at 12 weeks postoperatively.

### 8.5. Complications

Pin migration is a common complication following CRPP of proximal humerus fractures with potentially devastating complications [58]. Previous studies have demonstrated that threaded pins placed into the proximal humerus can migrate and cause neurovascular injury to major vascular or intra thoracic structures [51,58]. Close follow-up of these patients is mandatory and pins should be removed if there is any radiographic evidence of loosening.

There is an increased risk of infection if pins are left protruding through the skin. Superficial infections are typically treated with pin removal, local wound care, and oral antibiotics. However, deeper infections, though less common, can lead to significant complications including osteomyelitis [41].

Reported union rates following CRPP of proximal humerus fractures are high, likely secondary to minimal soft tissue dissection and devascularization. Loss of reduction and malunion more commonly occur than nonunion [41]. Varus angulation of the articular surface and posterosuperior displacement of the greater tuberosity are the most common malunions, which can lead to subacromial impingement, pain, and loss of function [41]. There is a higher risk of early pin loosening and loss of fixation in patients with osteoporosis and increased age.

The risk of avascular necrosis (AVN) is largely determined by the injury pattern itself [12]. The results of Kralinger et al. suggest that the rate of AVN was lower in patients treated with CRPP when compared with ORIF [59]. However, the findings of Kralinger et al. may be the result of selection bias as more severe fracture patterns, which have an increased risk of AVN, are more commonly treated with ORIF. Development of AVN following CRPP of proximal humerus fractures has been previously reported [41]. AVN may take up to 2 years following injury to develop and patients should be followed at regular intervals for that length of time.

Neurovascular injury is uncommon during CRPP of proximal humerus fractures [41]. In cadaveric studies investigating percutaneous pinning of the proximal humerus, injuries to the axillary nerve [60], cephalic vein, and biceps tendon [57] were noted. The results of the studies suggest that neurovascular structures are at risk during CRPP and thorough anatomical knowledge is mandatory.

### 8.6. Outcomes

The results of proximal humerus fracture CRPP have been favorable. Jaberg et al. showed that, for 48 CRPP patients included with an average of 3 years of follow up, 70% achieved a good or excellent result [61]. Similarly, Fenichel et al. reported 70% good or excellent results and an average constant score of 81 (scale 0–100) in 56 patients treated with CRPP [52]. Likewise, Keener et al. reported good or excellent outcomes for 27 proximal humerus fractures treated with CRPP, with an average of 35 months of follow up [56]. In a cohort of 27 patients, Resch et al. reported a mean constant score of 91 for three-part fractures and 87 for four-part fractures fixed with CRPP [55].

## 9. Open Reduction and Internal Fixation

Patients with proximal humeral fracture patterns that may benefit from operative fixation but do not meet criteria for closed reduction percutaneous are candidates for open reduction internal fixation. Patients with un-reconstructable head splitting fractures, fractures devoid of all soft tissue attachments [48], severe valgus impacted fractures with disruption of the medial periosteal hinge [12,48,49], and displaced multi-part fractures with delayed presentation may benefit from primary arthroplasty [27].

## 10. Surgical Approach

### 10.1. Patient Positioning

For operative fixation patients are typically positioned in the beachchair or supine position. The patient should be positioned with the head immobilized and the shoulder corner pushed away from the operative side in order to enable C-arm fluoroscopy access from the opposite side of the table.

### 10.2. Surgical Technique

Traditionally the deltopectoral approach has been the most widely utilized approach for fixation of proximal humerus fractures. The deltoid-split is an alternative approach that offers improved access proximally to the tuberosities and articular surface for reconstruction. During a deltopectoral approach the surgeon can improve visualization of a retracted tuberosity fragment by passing heavy stay sutures through the rotator cuff and drawing it anteriorly.

There is increased risk of intraoperative axillary nerve injury or late deltoid insertion pull off with the deltoid-split approach [62]. It also relies on indirect visualization of the fracture reduction especially at the medial calcar. When performing a deltoid-split approach a 5 cm longitudinal incision is made in line with the arm along the posterior border of the clavicle. The deltoid is split bluntly at the raphe between the anterior and middle thirds of the deltoid producing an upper “window” exposing the entire proximal humerus. Next care is taken to identify the anterior terminal branch of the axillary nerve and its accompanying vessels. After protecting the neurovascular bundle, the split can be extended distally to produce a lower “window” exposing the proximal portion of the humeral diaphysis. Axillary nerve injuries and injuries to the deltoid insertion have been reported but are uncommon in clinical practice [44,48,63,64,65]. Compared with the deltopectoral approach, the deltoid split approach offers better access to the greater tuberosity and posterior fracture patterns, as well as the lateral aspect of the humerus for plating [63,64]. The deltoid split approach can also be very useful in valgus impacted proximal humerus fractures where the lesser tuberosity is greatly displaced.

### 10.3. Fracture Reduction

The goal of operative fixation is to achieve an anatomic reduction of the key fracture fragments. Fracture reduction should be performed in a logical sequence. If the humeral head is dislocated it should be reduced first under direct visualization. The surgeon can gain access to the empty glenoid through a rotator interval arthrotomy. In anterior fracture dislocations the humeral head is typically engaged on the anterior glenoid rim, and on the posterior glenoid rim in posterior fracture dislocations. The surgeon must first disimpact the humeral head from the glenoid; this can be performed with a periosteal elevator or osteotome. Following dis-impaction the glenohumeral joint should then be reduced and the articular surface can be pinned after achieving acceptable alignment and angulation. At this point bone grafting procedures may be performed if indicated. The surgeon then can choose to either reduce the tuberosities to the head anatomically or reduce the head to the proximal shaft. In order to help facilitate reduction of the tuberosities heavy stay sutures may be passed through the bone-tendon interface to help with tuberosity manipulation. Several technical tricks may help the surgeon achieve an anatomic reduction: Kirschner wires can be inserted into fracture fragments and used as joysticks to manipulate the fragments, unicortical plates can be used as a preliminary reduction tool, a bump in the axilla can help lateralize a medially displaced shaft, and you can even pin the head to the glenoid. 

Often displaced proximal humeral fractures are unstable following reduction due to poor bone quality and comminuted fracture patterns. Adjuvant techniques can be helpful to improve construct stability and prevent early displacement. Typically the cancellous proximal humeral metaphyseal bone is impacted in three- and four-part fractures [26,48]. These defects can be filled with morselized autograft or with allograft [27,66,67]. Posteromedial calcar comminution of the surgical neck is frequently encountered in displaced surgical neck fractures with varus angulation [68]. Several studies have highlighted posteromedial calcar comminution as a risk factor for varus collapse of the surgical neck as a result of posteromedial instability [27,45,65,69,70]. The use of a locking plate in which screws are inserted along the calcar in a low position within the head to buttress the area of comminution, or a fibular strut graft have been shown to be helpful in restoring stability of fractures with calcar comminution [27,65,70]. The fibular strut graft helps restore stability by bridging across the area of comminution and substitutes for the compromised posteromedial buttress. In an elderly patient with severe comminution it can occasionally be appropriate to impact the shaft within the head to help restore stability by producing “bayonet” apposition of the shaft within the head fragment [27]. Heavy stay sutures through the tuberosities can be passed through eyelets in the plate, which creates a tension-band construct that counterbalances the deforming forces of the rotator cuff.

### 10.4. Fracture Fixation

**Figure 1:**
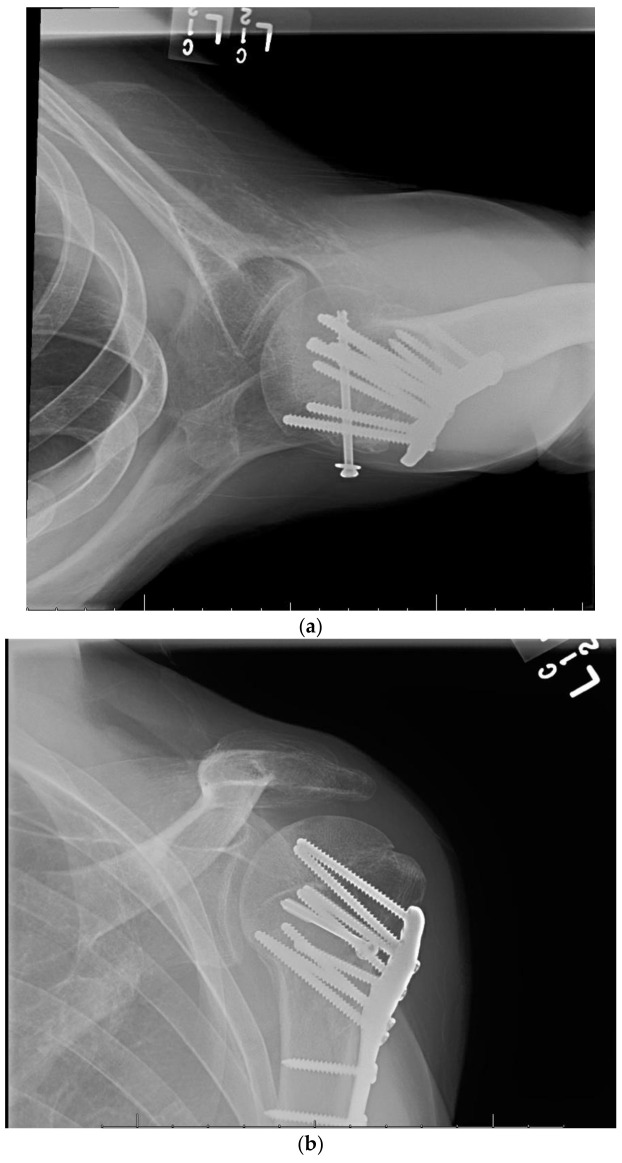

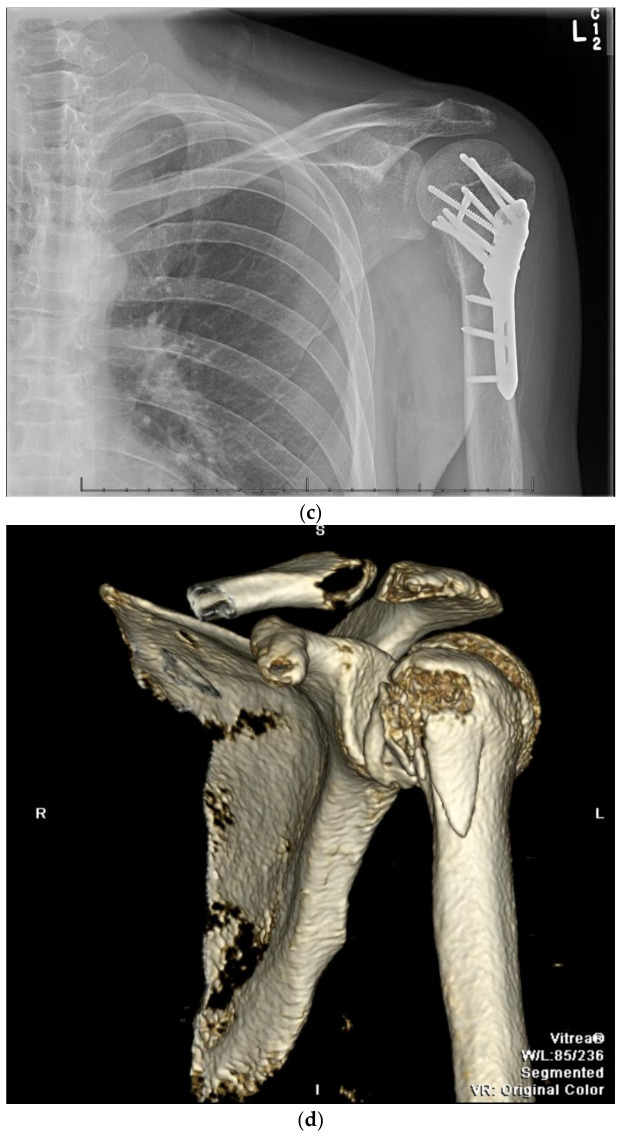

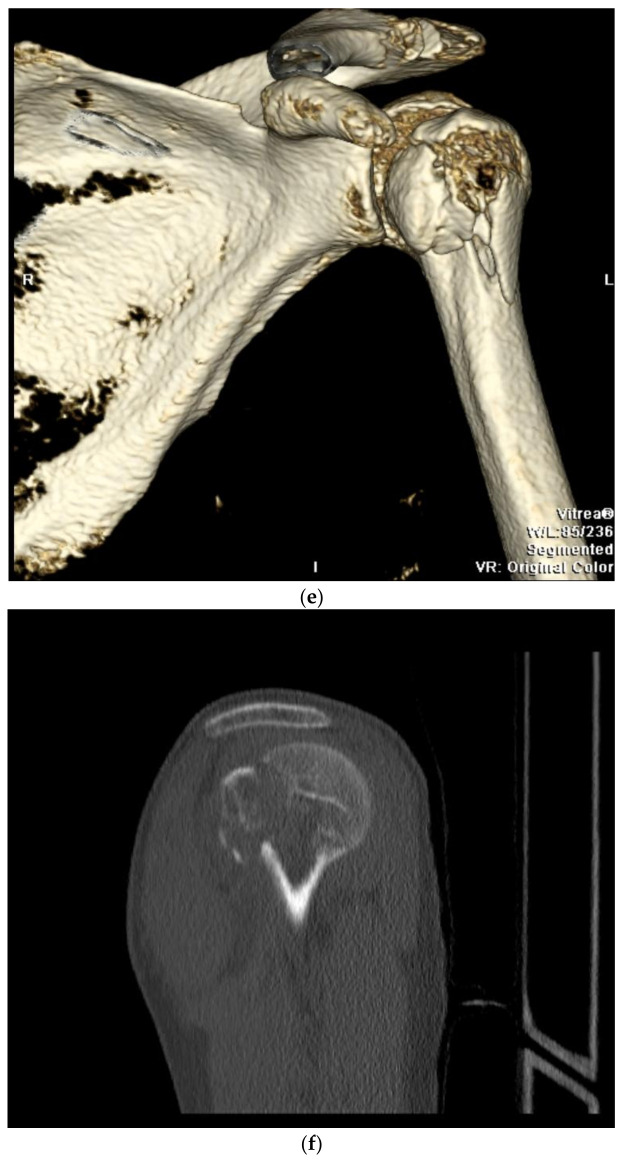

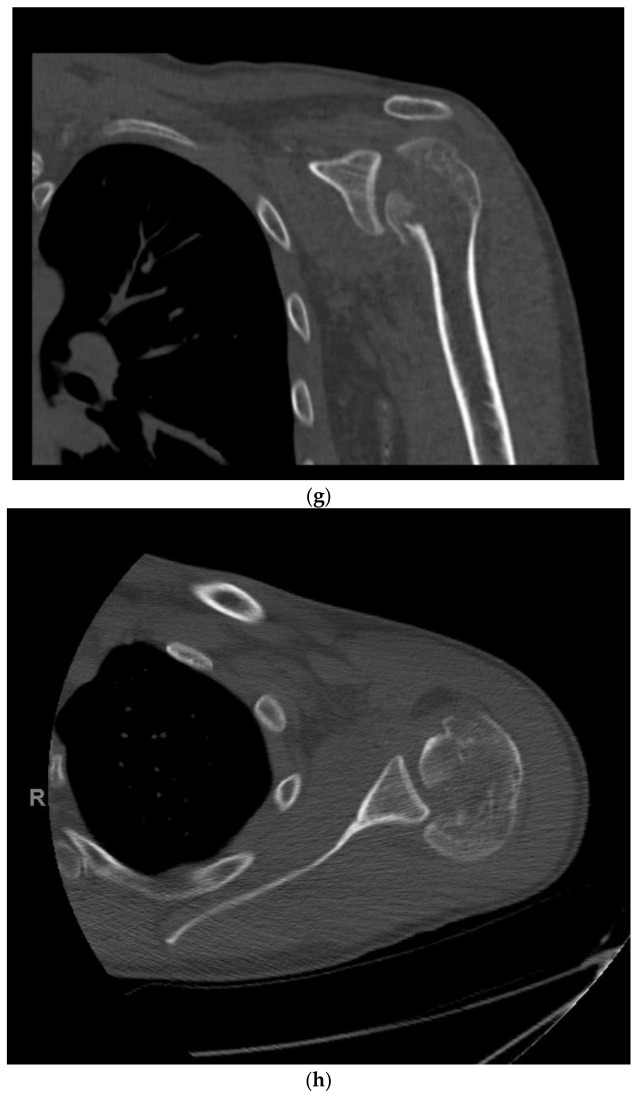
(**a**–**h**) Proximal Humeral Fracture Dislocation Treated with Open Reduction Internal Fixation.

The plate should be positioned on the lateral aspect of the proximal humerus posterior to the bicipital groove, in order to prevent damage to the long head of biceps and arcuate artery, and it must also be caudal to the cranial-most aspect of the greater tuberosity to avoid subacromial impingement. The subchondral bone beneath the articular surface is predictably dense, however bone mineral density does progressively decreases towards the center of the humeral head and extending distally into the metaphysis [71]. The surgeon should attempt to place screws in the calcar and inferior aspect of the humeral head when possible as the anterosuperior quadrant has the weakest bone [62,72,73]. The medial calcar screws are the most important screws in the construct and should drive the cranial/caudal plate positioning. In order to avoid joint penetration screws should be placed 5 to 10 mm from the articular surface [27]. Screws placed superiorly are at greatest risk of joint penetration. Open reduction and internal fixation of a proximal humeral fracture dislocation is shown in Figure 1.

### 10.5. Postoperative Management

Postoperative rehabilitation regimens vary between surgeons but typically follow the same general principles. Patients are immobilized 2–4 weeks postoperatively depending on the stability of fracture fixation. Immediate active elbow, wrist, and hand range of motion is permitted. Pendulum exercises can be started immediately. Gradually passive shoulder range-of-motion exercises are introduced following sling removal. Active-assisted range of motion can be initiated followed by strengthening exercises at 12 weeks postoperatively.

## 11. Open Reduction Internal Fixation with an Intramedullary Nail

Historically intramedullary nails (IMN) were uncommonly utilized for fixation of proximal humerus fractures due to concerns regarding rotator cuff morbidity, iatrogenic fracture, and proximal screw migrations [74]. However modern nails include improved locking mechanisms for the proximal screws with fragment specific fixation as well as a straight geometry that allows for insertion of the nail medial to the insertion of the rotator cuff. These improved IMN designs has led to a resurgence of interest in utilizing these implants for fixation of proximal humerus fractures [75]. Intramedullary nails may hold advantages over proximal locking humerus plates in select patients with proximal humeral fractures including a smaller approach with less risk of blood loss.

## 12. Surgical Approach for ORIF with an Intramedullary Nail

### 12.1. Patient Positioning

The patient should be placed in the same position as discussed above in ORIF.

### 12.2. Surgical Technique

There are several described techniques for the approach to intramedullary nailing of proximal humerus fracture. For the authors preferred technique a small incision is made in line with the anterior border of the acromion extending distally. The raphae between the middle and anterior thirds of the deltoid are split bluntly to access the subdeltoid space. A limited bursectomy can be performed to expose the insertion of the rotator cuff on the greater tuberosity. The rotator cuff tendon should be inspected for any associated tearing. Often a traction stitch through the rotator cuff musculotendinous junction can be utilized to help mobilize the head fragment and expose the supraspinatus muscle bed. Care is then taken to make a longitudinal incision through the supraspinatus muscle exposing the articular margin of the humeral head, which is the insertion point for a straight humeral IMN. The long head of the biceps tendon is routinely encountered anterior to the start point for the IMN and should be protect. In contrast, the appropriate start point for a curved IMN is more lateral, which can potentially violate the tendinous insertion of the rotator cuff on the greater tuberosity.

### 12.3. Fracture Reduction

Reduction can be obtained through the various closed reduction techniques discussed above. Again, a traction stitch through the supraspinatus tendon can be helpful in correcting humeral head varus. Kirschner wires can be inserted into fragments and used as joysticks to aid with reduction.

### 12.4. Fracture Fixation

**Figure 2:**
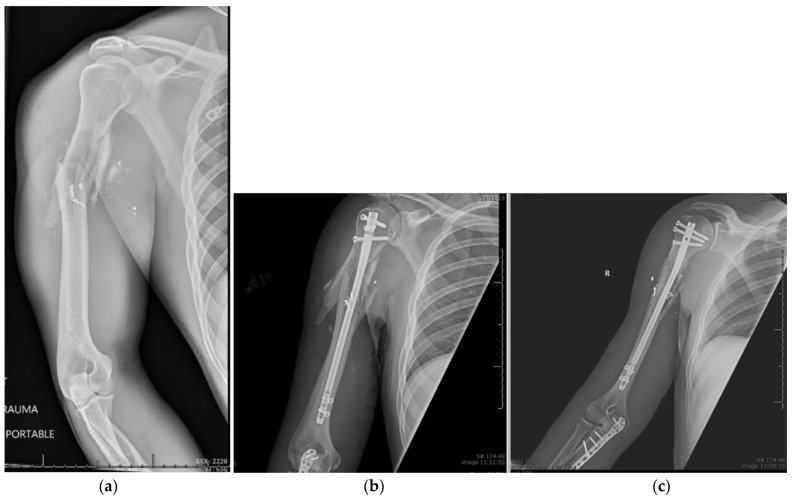
(**a**–**c**) Ballistic Proximal Humeral Shaft Fracture Treated with Intramedullary Nail Fixation.

In order to access the start point for a humeral IMN, the arm must be positioned in slight extension and adduction. Once the reduction is confirmed on fluoroscopy, a guide pin is inserted in line with the axis of the humeral shaft. Every system has its particular technique but all require a correctly placed guide wire followed by a reamer or a handheld awl to create a proximal opening for passing the nail. Longer nails may require diaphyseal reaming over a longer guidewire. The humeral IMN is then inserted under fluoroscopy and care is taken to prevent iatrogenic fracture. The proximal end of the humeral IMN must be placed at least 5 mm beneath the cortex of the humeral head; no protrusion of the nail can be tolerated. In order to lock the nail with the correct trajectory, the aiming arm should be rotated anteriorly approximately 25 degrees to match the retroverted axis of the humeral head. Unicortical locking screws are then placed in the proximal portion of the nail to prevent intra-articular penetration, and bicortical distal locking screws are placed distally. After confirming fracture reduction and nail position, continuous fluoroscopy should be utilized while rotating the arm to confirm that no proximal screws penetrate the glenohumeral joint. Rotator cuff tears can then be repaired with transosseous sutures or suture anchors through the incision. The supraspinatus muscle fibers should be repaired side-to-side as well as the deltoid raphae. Ballistic proximal humeral shaft fracture treated with intramedullary nail fixation is shown in Figure 2.

### 12.5. Postoperative Management

Patients are typically immobilized for 6 weeks postoperatively in an abduction pillow sling. Immediate active elbow, wrist, and hand range of motion is permitted as well as passive shoulder ROM. Pendulum exercises can be started immediately. Active-assisted and full active range of motion exercises can begin 4–6 weeks after fixation depending on fracture morphology, with strengthening exercises beginning at approximately 12 weeks postoperatively.

### 12.6. Complications

The most common complications of open reduction and internal fixation of proximal humeral fractures are post-traumatic arthrofibrosis, nonunion, malunion, osteonecrosis, early fixation failure, and infection.

Post-traumatic arthrofibrosis following ORIF of proximal humeral fractures has a good prognosis if the fracture heals. Clinically patients present similar to primary adhesive capsulitis with painful loss of passive shoulder range of motion [76]. The majority of patients will typically respond to physical therapy. If there is a plateau in recovery at 6 months post op then manipulation under anesthesia may be required. If manipulation fails to restore acceptable range of motion, then, under the same anesthesia event, arthroscopic rotator interval and capsular release should be performed [27,77]. There is increased risk of peri-implant fracture during manipulation under anesthesia, thus care should be taken to avoid this complication.

Nonunion can occur secondary to a mechanically unstable fracture reduction and fixation or as a consequence of poor surgical technique. Patients often presents with pain, decreased range of motion, and loss of function with plain films that confirm bone resorption and lack of healing. Revision ORIF is usually not feasible and revision to an arthroplasty is recommended if symptoms are debilitating [27]. In contrast, malunion is generally well tolerated in elderly patients with limited functional demands. However, younger patients can develop debilitating symptoms from malunion causing poor shoulder function, subacromial impingement, and tear of the rotator cuff. Corrective osteotomies or conversion to arthroplasty can be considered for young patients with severe symptom secondary to malunion.

Osteonecrosis has been reported in up to 15% of patients following ORIF for proximal humeral fractures [27]. Fracture dislocations and displaced multipart fractures are at higher risk of osteonecrosis secondary to disruption of the humeral head end arterial blood supply [12,27,44,48]. Osteonecrosis can also occur secondary to poor surgical technique with excessive soft tissue stripping. Patients clinical presentation should be the major determinant in therapeutic decision making. Some Patients may present postoperatively with radiographic evidence of osteonecrosis and minimal symptoms, and these people are likely tobe amenable to nonoperative treatment [78]. However, the majority of patients with proximal humeral osteonecrosis will develop symptomatic advanced collapse [79]. Conversion to arthroplasty should be considered for patients with severe symptoms and advanced collapse.

Infection is relatively uncommon following proximal humeral ORIF secondary to rich vascularity and good soft tissue coverage [27]. Superficial infections typically resolve with oral antibiotic therapy. Deep infections with stable hardware should be treated with incision and debridement, preservation of fixation, and antibiotic therapy. If the infection is refractory to hardware retention and prolonged antibiotic therapy, then a more radical debridement with implant removal should be performed with delayed reconstruction.

### 12.7. Outcomes

Gomberwalla et al. performed a meta-analysis on data from 12 studies comparing open reduction and internal fixation of proximal humerus fractures to arthroplasty for treatment of 3- and 4-part fracture patterns. Open reduction internal fixation demonstrated significantly higher constant scores than arthroplasty [80]. However, the studies included exhibited significant heterogeneity limiting the study’s conclusions. Constant scores were observed to decreased significantly with severity of fracture, increasing age, and osteonecrosis [80]. 

Mayr et al. randomized patients presenting with a proximal humeral fracture to fixation with either an intramedullary nail or a locking plate. At 12 months follow up, the authors found no significant difference in pain, range of motion, or overall complication rate [81]. There was no difference in functional outcome assessed by the Constant score, however DASH scores were slightly lower in the intramedullary nail group at 12 months [81]. Loss of reduction and screw cut out was significantly lower in the intramedullary nail group [81]. When comparing straight to curvilinear intramedullary nails Lopiz et al. demonstrated no difference in Constant score between the constructs [82]. However, straight IMN demonstrated significantly lower incidence of rotator cuff dysfunction and incidence of screw loosening [82].

## 13. Reverse Total Shoulder Arthroplasty

Reverse total shoulder arthroplasty (RSA) is another treatment option for three and four-part proximal humerus fractures. RSA was initially developed to treat glenohumeral arthritis in the rotator cuff deficient shoulder. The implant design creates a more constrained joint that moves the center of rotation of the glenohumeral joint medial and distal to improve the mechanical advantage of the deltoid, thus providing function to rotator cuff deficient shoulders. Since the 1990’s, RSA have become increasingly utilized for treatment of proximal humerus fractures. Advantages of RSA in the setting of proximal humerus fracture are that the outcome is less dependent on healing of the tuberosities (as is the case anatomic shoulder arthroplasty), or perfect reduction of the tuberosities. Furthermore, the biomechanical design of the RSA guards against underlying chronic or acute rotator cuff deficiency. Some studies have demonstrated better functional outcomes and pain relief with RSA when compared ORIF and hemiarthroplasty [83,84].

In patients with three- and four-part proximal humerus fractures, RSA is one of three main surgical options. Reverse total shoulder arthroplasty should be considered in elderly patients with un-reconstructable head-split fractures, irreducible cartilage defects, and patients at increased risk of failure of tuberosity healing [85,86]. Contraindications include deltoid insufficiency and permanent axillary nerve deficits, as well as acromial/scapular fractures that would de-tension the deltoid and glenoid fractures that preclude stable baseplate placement.

## 14. Surgical Approach

### 14.1. Patient Positioning

For RSA of proximal humerus fractures, patients are typically positioned in the “beachchair” position. The patient should be positioned with the head immobilized and the shoulder corner supporting the scapula.

### 14.2. Surgical Technique

For RSA of proximal humerus fractures patients are typically positioned in the “beachchair” position. The patient should be positioned with the head immobilized and the shoulder corner supporting the scapula.

### 14.3. Surgical Technique

The deltopectoral approach is commonly used for RSA. Surgeons most familiar with the approach in arthritis patients must be cognizant of how acute and subacute fractures can alter the approach. Care must be taken during dissection to avoid injuring the neurovascular structures due to the distorted position of bony landmarks and native anatomy. Once the approach is complete and the fracture is identified, the long head of the biceps tendon should be identified and cut for tenodesis to the upper border of the pectoralis major tendon.

When either or both tuberosities are the surgeon should work to clean the tuberosities and tag them with stay sutures placed into the rotator cuff musculotendinous junction. If there is no fracture, the tuberosities should be osteotomized to a manageable size, which allows for preservation of the rotator cuff musculature. The humeral head is then removed and saved for bone grafting. Attention is then shifted to the glenoid. When treating proximal humerus fractures with RSA, in general, glenoid visualization is less difficult when compared with primary arthroplasty due to fracture of the humeral neck. After exposure of the glenoid, the glenoid is then cleaned and prepared. A large glenosphere, is placed according to the specifications of the implant manufacturer.

After placing the glenosphere, the surgeon then shifts attention back to the humerus. The surgeon should attempt to preserve as much bone stock as possible while reaming the humeral canal. Press-fit fixation is typically the goal, however cemented stems are often required due to (1) the fracture pattern and (2) poor bone quality. Pilot holes are drilled in the humeral shaft for tuberosity fixation. The humeral stem is then trialed. Care should be taken to retrovert the stem 10–30 degrees. The humeral components are then placed in accordance with manufacturer specifications. It is unclear whether cemented or uncemented fixation for RSA is superior [87]. Humeral components with head-neck angles of 135° and 155° are available for use. Those with 135° are more physiologic and decrease tension on tuberosity healing.

**Figure 3:**
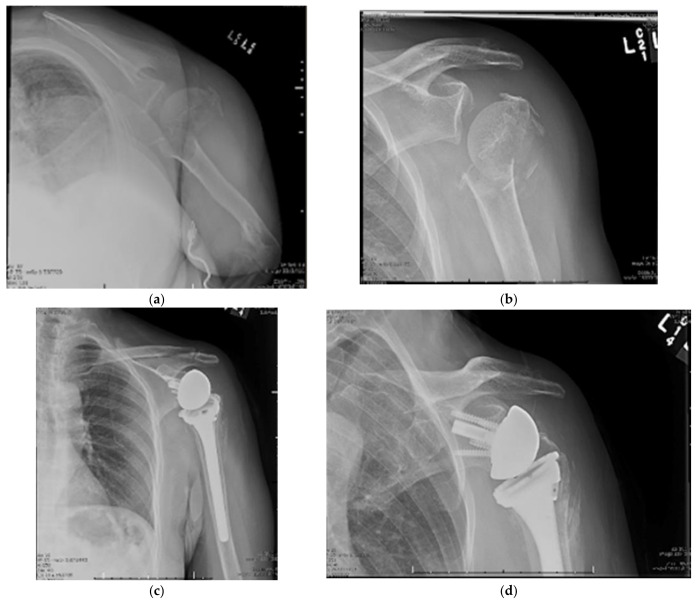
(**a**–**d**) Neer Four-Part Proximal Humerus Fracture Treated with Reverse Total Shoulder Arthroplasty.

After placing the humeral component the greater and lesser tuberosities are then reduced and tied down to their corresponding pilot holes using high strength suture [83]. Cancellous autograft can be used to address bony defects. A hybrid form of cement and cancellous autograft impaction technique has been described as well [88]. There are fracture-specific implants with collars, metaphyseal hydroxyapatite coating, and bone grafting windows [89]. Post-operatively, patients are immobilized in a sling for 2–3 weeks at which time physical therapy is initiated, first focusing on range of motion, and then strengthening. Proximal humeral fracture treated with RSA is shown in Figure 3.

### 14.4. Outcomes

When compared with hemiarthroplasty, RSA for treatment of proximal humeral fractures has been shown to have improved Constant scores, better active forward flexion, and better abduction [84,90,91,92].

Two recent meta-analyses attempt to compare outcomes of operative treatment for proximal humeral fractures across a number of studies and interventions. Chen et. al. demonstrated that RSA results in better functional outcomes when compared with both hemiarthroplasty and ORIF. The authors also concluded that RSA had the lowest rate of complications and additional surgery [93]. Another more recent meta-analysis, including only randomized control trials, found superior outcomes in RSA compared to hemiarthroplasty [94]. A third meta-analysis of only randomized controlled trials also supports RSA’s superiority when compared with hemiarthroplasty [95].

There have also been a number of studies and meta-analyses directly comparing RSA to ORIF and HA. Suroto et al. found that RSA had lower rates of revision surgery, better forward flexion and abduction, and superior functional outcome measured by Constant Scores when compared with ORIF [91]. Perhaps the highest quality most recent study, the DelPhi study, demonstrated Constant scores that were 13.4 points higher in the RSA group than the ORIF group [92]; however the editors cautioned that follow-up was limited to two years [96].

In recent literature RSA consistently performs better than hemiarthroplasty with more reproducible functional outcomes and better recovery of flexion and abduction, but worse internal and external rotation [90]. Total complication rates are higher in RSA, however the reoperation rate is similar to hemiarthroplasty, and the rate of revision surgery is lower in RSA than in HA [90].

There is limited data on long-term outcomes of RSA, especially in proximal humerus fracture patients. Long-term data available in the arthritis population shows sustained functional outcomes and implant survival (Table 1).

Bacle et al. published 10-year outcomes of patients treated for proximal humerus fracture with RSA. Of the patients with appropriate follow-up, Constant scores were significantly lower when compared to their medium-term scores at 2-years. Constant score were still significantly higher than pre-operative levels. The authors also found that only 10% of complications occurred in the period between 2- and 10-year follow-up, and implant survival was 93% at 10 years [97].

RSA has been shown to have improved outcomes in patients over 70 years old when compared with hemiarthroplasty and ORIF, however there remains debate for the optimal age cutoff at which the risk of RSA longevity outweighs the risks of failure of the other surgical modalities. Long-term outcome data from studies of patients who underwent RSA for rotator cuff tear arthropathy demonstrate acceptable long-term implant survival, but there is insufficient longevity data in the fracture population. There also remains a lack of evidence on the long-term cost-effectiveness of RSA compared to alternative treatment options given the higher cost of implants. Medium term data has shown RSA to be cost-effective compared with alterative treatments [98,99]. Despite the lack of long-term data, RSA is a viable treatment option for the correct fractures in the elderly patients.

## 15. Hemiarthroplasty

Humeral head replacement was presented as the logical solution to ORIF’s inability to effectively treat humeral head excision [100]. Hemiarthroplasty (HA) used to be the primary method for treating complex proximal humerus fractures. However, due to the rise of new locking plate technology and the reverse total shoulder arthroplasty, indications for HA have become more limited in recent years [16,101,102]. RSA has been found to produce more consistent outcomes in patients over 70 years of age while prosthetic shoulder replacement is rarely recommended in patients younger than 60 years old [16,103]. Thus, HA’s indications are limited to middle-aged patients with 3-part head splitting fractures or four-part fracture dislocations [16].

### 15.1. Patient Positioning

For shoulder hemiarthroplasty of proximal humerus fractures, patients are typically placed in the beach chair position, with the head of the bed elevated to 45° and the injured extremity free [104].

### 15.2. Surgical Approach

**Figure 4:**
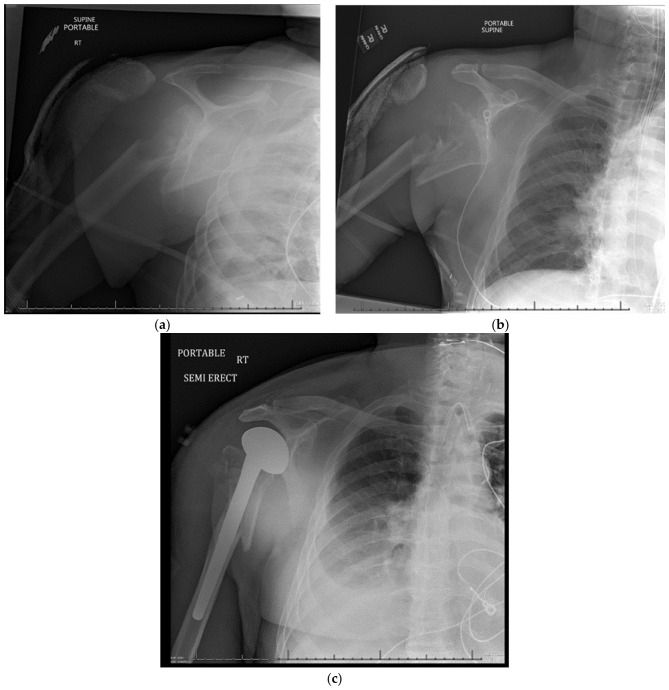
(**a**–**c**) Neer Four-Part Proximal Humerus Fracture Treated with Hemi Arthroplasty.

The deltopectoral approach is the most common approach used, as it provides the greatest humeral exposure [16,100,102,105]. An incision should start just proximal and medial to the coracoid process and extend distally down to the anterior aspect of the arm. The cephalic vein should be identified and retracted, exposing the interval between the deltoid and pectoralis major muscles. A Longitudinal incision of the clavipectoral fascia is made to expose the tuberosities and rotator cuff musculature. The tuberosities are typically tagged with heavy sutures, which allows them to be mobilized and retracted for visualization. Proper anatomic tuberosity healing is key for functional outcome. Moreover, selecting proper prosthetic head size and head height is crucial for the tuberosity healing process and for the overall result of this procedure. The “jigsaw method” is a reliable approach for determining the best height and prosthetic version. For trial prosthetic reduction, the humeral head is reduced onto the glenoid and the tuberosities are pulled into position. Fluoroscopic images are then taken. Alternatively, In the case of press-fit stems trial reductions can be performed to obtain correct height and tuberosity reduction. Strong and secure tuberosity fixation is paramount for a successful outcome in shoulder hemiarthroplasty. For low quality/osteoporotic bone, cementation of the stem reduces the chance of intraoperative fracture and later subsidence [17]. Once the reduction is deemed acceptable, the final implant is inserted. Proximal humeral fracture treated with hemiarthroplasty is shown in Figure 4.

### 15.3. Postoperative Management

Postoperatively, sling immobilization typically lasts 2–4 weeks [106]. Early passive ROM exercises are typically started 1 to 3 days after surgery [102,106]. Strength of tuberosity fixation should play a role in determining forward elevation and internal rotation limits during this time. Active ROM is typically initiate at 6 to 8 weeks after surgery, with motion limited to neutral rotation and 90 degrees of forward flexion. Isometric deltoid and internal and external rotation strengthening can begin at 8 to 10 weeks, but resistive strengthening exercises are not recommended for a least 3 months post-operatively.

### 15.4. Complications

Complications for shoulder hemiarthroplasty include infection, intraoperative fracture, tuberosity nonunion/malunion, stiffness, rotator cuff disfunction, heterotopic bone, glenoid erosion or arthrosis, and instability [16,17,101,104,107]. Kontakis et al. found the incidence of superficial and deep infection to be 1.55% and 0.64%, respectively, in shoulder HA [108]. For superficial infection and acute deep infection, treatment involves irrigation and debridement followed by culture-guided antibiotics [104]. Chronic deep infection may necessitate implant removal, followed by the placement of an antibiotic spacer with prolonged IV antibiotic therapy, and delayed reimplantation [104]. 

Glenoid arthrosis is seen more often in younger and middle-aged patients who place their shoulder at a higher demand [106,109]. Wear of the glenoid cartilage correlates with lower functional outcome and may lead to conversion to TSA. 

Complications related to tuberosity healing are the most common type of complication following shoulder hemiarthroplasty. Boileau et al. found that 50% of HA procedures result in tuberosity malposition [110]. Rotator cuff dysfunction is often correlated with tuberosity complications, and both are detrimental to the overall functional outcome of the procedure. Choosing proper prosthetic version and height is crucial for preventing these complications [16]. Tuberosity and rotator cuff complications often require revision to a reverse total shoulder to correct the deficiencies. 

Injury to axillary nerve is the most common neurological complication for shoulder HA, but injury to other branches of the brachial plexus is also possible [108,110]. In Boileau et al.’s analysis, 4% of shoulder hemiarthroplasty patients had nerve injury complications [110]. Most of these nerve injuries are transient and resolve after observation and therapy [104].

### 15.5. Outcomes

In 1970, Neer et al. found favorable outcomes for shoulder hemiarthroplasty in four-part fractures, determining that HA was superior to open and closed reduction methods for these types of fractures (Neer 1970). Since then, studies on shoulder HA outcomes have been inconsistent. As discussed earlier, Boons et al. found no significant difference in functional outcome between shoulder HA and non-operative treatment of proximal humerus fractures at 3 month and 1 year follow-up [111]. In contrast, a randomized trial by Olerud et al. found that shoulder HA led to superior quality of life scores than non-operative treatment for elderly patients with 4-part fractures [36]. However, a randomized trial by Laas et al. found shoulder HA to have inferior functional outcome when compared with RSA for elderly patients with dislocated 3-par and 4-part fractures [103]. Similarly, a retrospective study by Solberg et al. found that locked plate osteosynthesis had better functional outcome than shoulder HA in 3-part and 4-part proximal humerus fractures, as measured by mean Constant score [112]. Overall, several studies have found that shoulder hemiarthroplasty leads to pain relief, but poor or unpredictable outcomes for range of motion [101,113,114]. The advantages and disadvantages of operative methods for treating proximal humerus fractures are listed in Table 2.

## Figures and Tables

**Table 1:** Long-Term Outcomes and Complications of RSA.

10 Year Implant Survival	87–93%
15 Year Implant Survival	79–92%
Constant Score	57–62%
Forward Flexion	101–139°
Abduction	86–135°
External Rotation	11–48°
Prosthetic Joint Infection	2.4%
Grade III/IV Scapular Notching	29.4%
Acromial/Scapular Fracture	2.6%
Loosening/Instability	1.3%

**Table 2:** Advantages and Disadvantages of Operative Methods for Treating Proximal Humerus Fractures.

Operative Method	Advantages	Disadvantages
Closed Reduction Percutaneous Pinning	Less soft tissue stripping Minimally invasive Lower rates of avascular necrosis Higher union rates Decreased scar formation	Technically challenging procedure Loss of reduction and malunion Pin migration Increased risk of superficial infection
Open Reduction Internal Fixation	Earlier return to function compared with closed reduction percutaneous pinning	Post-traumatic arthrofibrosis Avascular necrosis Worse functional outcomes compared to reverse total shoulder arthroplasty
Intramedullary nailing	May hold advantages over proximal locking humerus plates, including a smaller approach with less risk of blood loss	Concerns of rotator cuff morbidity, iatrogenic fracture, and proximal screw migrations
Reverse Total Shoulder Arthroplasty	Clinical outcome is less dependent on healing or perfect reduction of the tuberosities Quicker recovery Fewer restrictions on rehabilitation Better functional outcomes compared to both open reduction internal fixation and hemi arthroplasty	Limited data available on long term outcomes of reverse total shoulder arthroplasty Higher complication rate than hemi arthroplasty Dislocation Glenoid fracture Scapular notching, loosening
Hemi Arthroplasty	Lower complication rate than reverse total shoulder arthroplasty Relatively favorable outcomes for pain relief	Technically demanding High rates of malposition and tuberosity migration Less consistent functional outcomes compared to reverse total shoulder arthroplasty

## Data Availability

Not applicable.
